# Prospective relations between maternal emotional eating, feeding to soothe, and infant appetitive behaviors

**DOI:** 10.1186/s12966-021-01176-x

**Published:** 2021-08-11

**Authors:** Chelsie D. Temmen, Leah M. Lipsky, Myles S. Faith, Tonja R. Nansel

**Affiliations:** 1 Social and Behavioral Sciences Branch, Division of Intramural Population Health Research, Eunice Kennedy Shriver National Institute of Child Health and Human Development, 6710B Rockledge Dr., MSC 7004, Bethesda, MD 20892 USA; 2 Department of Counseling, School, and Educational Psychology, Graduate School of Education, University at Buffalo – SUNY, 420 Bady Hall, Buffalo, NY 14250-1000 USA

**Keywords:** Infancy, Feeding to soothe, Appetitive behaviors, Eating behavior, Mother-infant relationships

## Abstract

**Background:**

Infant obesogenic appetitive behaviors are associated with greater infant weight and child obesity, yet little is known about maternal influences on infant appetitive behaviors. This study examines the relations between maternal eating behaviors, feeding to soothe, and infant appetitive behaviors in a longitudinal sample of United States mothers.

**Methods:**

Pregnant women were recruited in the first trimester (< 12 weeks) and followed through 1 year postpartum. Mothers reported their own eating behaviors (eating competence, restrained, emotional, and external eating) in pregnancy; feeding to soothe their infant at 2, 6, and 12 months postpartum; and their infants’ appetitive behaviors (enjoyment of food, food responsiveness, slowness in eating, and satiety responsiveness) at 6 months. Three path models were estimated to examine the direct relations of maternal eating behaviors with infant appetitive behaviors, the indirect relations of maternal eating behaviors with infant appetitive behaviors through feeding to soothe, and the longitudinal relations between feeding to soothe and infant appetitive behaviors.

**Results:**

Maternal eating behaviors and infant appetitive behaviors were directly and indirectly related in all three models. Greater maternal eating competence was related to greater enjoyment of food but was not related to feeding to soothe. Greater maternal restrained and external eating were not directly related to infant appetitive behaviors but were indirectly related to greater infant responsiveness to food through more frequent feeding to soothe. Additionally, several longitudinal relations between feeding to soothe behaviors and infant appetitive behaviors were present. More frequent feeding to soothe at 2 months was related to greater responsiveness to food at 6 months, which was then related to more frequent feeding to soothe at 6 months. Furthermore, greater satiety responsiveness, faster eating speed, and greater responsiveness to food at 6 months were related to more frequent feeding to soothe at 12 months.

**Conclusions:**

Maternal eating behaviors were related to infant appetitive behaviors directly and indirectly through feeding to soothe. Additionally, results suggest feeding to soothe and infant appetitive behaviors may be bidirectionally linked. These results underscore the need to examine how parental feeding behaviors are influenced both by parental eating behaviors and child appetitive behaviors throughout infancy.

**Trial registration:**

Clinicaltrials.gov. Registration ID – NCT02217462. Date of registration – August 13, 2014.

## Background

Nearly one-fifth of children ages 6–19 years in the United States have obesity [1], suggesting the importance of understanding early origins of obesity. Infant obesogenic appetitive behaviors including greater enjoyment of food, faster eating speed, greater food responsiveness, and lower satiety responsiveness are associated with greater infant weight and child obesity [2, 3], faster infant growth [4], and more interest in food stimuli than non-food stimuli [5]. Although appetitive traits are 53–84% heritable [6], a substantial proportion of variance in appetite is attributed to environmental factors, including parental influences. Yet little is known about parental influences on infant appetitive behaviors.

Feeding to soothe a child in distress has been related to higher BMI in infants and toddlers [7, 8]; however, the relationship of feeding to soothe with infant or child appetitive behaviors is unknown. Related studies indicate that maternal feeding to soothe is positively related to emotional overeating in toddlers [9], and that parent emotional feeding (feeding in response to the child’s emotions) is associated with greater food responsiveness in toddlers [10] and more emotional overeating in school-aged children [11, 12]. Most research on associations of food-specific parenting with child eating behaviors hypothesizes that child eating behaviors are directly or indirectly related to parenting, although scant experimental or longitudinal data are available to inform the direction of these relations [13]. Similarly, the relation between feeding to soothe and child eating behaviors may be bidirectional. Infants may learn to eat in response to non-hunger cues, leading to obesogenic appetitive behaviors (e.g., greater food responsiveness). Furthermore, children who already display obesogenic appetitive behaviors (e.g., faster eating speed or greater enjoyment of food) may reinforce feeding to soothe because of the children’s eagerness to eat. To date, however, no study has examined this potential bidirectionality.

Parental obesogenic eating behaviors (e.g., emotional eating, eating in response to external stimuli) and eating competence (i.e., a person’s comfort and flexibility with eating) [14] may also be related to child appetitive behaviors and parent feeding behaviors. Maternal emotional eating was positively related to children’s desire to eat [15] in early and middle childhood, but another study found no relation with eating speed or food responsiveness in preschool-aged children [16]. Additionally, maternal emotional eating and external eating were related to more emotional feeding [17, 18], although null findings have also been reported [19]. No studies to date have examined relations between maternal eating competence and infant appetitive behaviors. Further, relations between maternal eating behaviors and infant appetitive behaviors may be mediated by feeding to soothe. Mothers may choose to feed to soothe more frequently if they frequently feed themselves in response to stress and they expect their child will response similarly to food when distressed. This frequent feeding to soothe driven by maternal obesogenic eating may then acclimate infants to more obesogenic eating behaviors. In preschool-aged children, greater maternal pressure to eat mediated the relationship of maternal external eating with picky eating [15], and feeding to soothe mediated the relations between maternal responsive parenting and emotional eating [9]. However, it is unknown whether feeding to soothe mediates the relations between maternal eating behaviors and infant obesogenic appetitive behaviors.

This study examined three main questions in a longitudinal study of mother-infant dyads: (a) how are feeding to soothe and maternal obesogenic eating behaviors prospectively related to infant obesogenic appetitive behaviors, (b) is there a reciprocal relationship between infant appetitive behaviors and maternal feeding to soothe, and (c) does feeding to soothe mediate relations of maternal obesogenic eating behaviors with infant appetitive behaviors? We hypothesized that feeding to soothe would be related to infant obesogenic appetitive behaviors (i.e., greater enjoyment of food, faster eating speed, greater food responsiveness, and lower satiety responsiveness). Additionally, we hypothesized that more maternal feeding to soothe would predict stronger infant obesogenic appetitive behaviors, and stronger infant obesogenic appetitive behaviors would predict more feeding to soothe. We also hypothesized that maternal obesogenic eating behaviors (i.e., more emotional, external, and restrained eating and less eating competence) would be positively related to infant obesogenic appetitive behaviors indirectly through increases in feeding to soothe.

## Methods

### Participants and procedures

Data for this secondary data analysis come from the Pregnancy Eating Attributes Study (PEAS), a prospective study of dietary intake and weight change in 458 pregnant women located in a single metropolitan area in North Carolina, United States. Women were recruited in the first trimester of pregnancy (≤ 12 weeks gestation) and followed through 1 year postpartum. Mothers were considered for inclusion if they had a confirmed uncomplicated singleton pregnancy ≤12 weeks gestation at enrollment, planned to deliver at the University of North Carolina Women’s Hospital and remain in the area for 1 year following delivery, and were age ≥ 18 and < 45 at screening with a BMI ≥ 18.5 kg/m2. Mothers were excluded from participation if they had been diagnosed with any medical condition or psychological disorder that may contraindicate participation in the study. Full details on the study methods are detailed in a previous publication [20].

Participants provided written consent for study participation. Study visits were conducted prenatally at baseline (< 12 weeks), 16–22 weeks, and 28–32 weeks gestation, and postpartum at approximately 2 months (4–14 weeks), 6 months (23–31 weeks), and 12 months (50–58 weeks). Participants completed self-administered measures through a secure online data collection system within a specified study visit windows. Data collection was completed in June 2018. All study procedures were approved by the University of North Carolina Institutional Review Board.

### Measures

Except where noted, all measures self-reported in pregnancy were collected once between baseline (< 15 weeks) and 27 weeks gestation.

#### Maternal eating competence

During pregnancy, mothers completed the 16-item ecSatter Inventory [14], which reflects four dimensions of eating competence (i.e., comfort, flexibility, and attitude around eating): “(a) attitudes about eating and about food; (b) food acceptance skills; (c) internal regulation skills; and (d) skills and resources for managing the food context and orchestrating family meals” [21]. Response options ranged from 0 (*never* or *rarely*) to 3 (*always*). Items were summed to create a total eating competence score (*α* = .88), with higher scores indicating greater eating competence.

#### Maternal obesogenic eating behaviors

During pregnancy, mothers completed the 33-item Dutch Eating Behavior Questionnaire [22], which measures restrained (10 items; *α* = .83; e.g., “how often do you refuse food or drink offered because you are concerned about your weight?”), emotional (13 items; *α* = .93; e.g., “do you get the desire to eat when you are anxious, worried, or tense?”), and external (10 items; *α* = .83; e.g., “if you see others eating, do you also have the desire to eat?”) eating behaviors. Response options ranged from 1 (*never*) to 5 (*very often*). The items in each subscale were averaged to create the three subscales, with higher scores indicating greater endorsement of restrained, emotional, and external eating behaviors.

#### Feeding to soothe

At each postpartum visit, mothers completed the 13-item Food to Soothe Questionnaire [7]. This measure asks mothers how often they used food to soothe their infants in various situations. Response options ranged from 1 (*never*) to 5 (*often*). Items were averaged to create total feeding to soothe scores at 2 months (*α* = .93), 6 months (*α* = .85), and 12 months (*α* = .83), with higher scores indicating more frequent feeding to soothe.

#### Infant appetitive behaviors

At 6 months postpartum, mothers completed the 17-item Baby Eating Behavior Questionnaire [23]. This questionnaire measures four appetitive constructs during the period of exclusive milk-feeding: enjoyment of food (4 items; *α* = .75; e.g., “my baby enjoyed feeding time”), food responsiveness (6 items; *α* = .80; e.g., “if given the chance, my baby would always be feeding”), slowness in eating (4 items; *α* = .62; e.g., “my baby sucked more slowly”), and satiety responsiveness (3 items; *α* = .39; e.g., “my baby got full up quickly”). Response options ranged from 1 (*never*) to 5 (*always*). The items for each subscale were averaged to create the subscales, with higher scores indicating greater enjoyment of food, greater food responsiveness, slower eating speed, and greater satiety responsiveness.

#### Demographic variables

At baseline, mothers reported sociodemographic characteristics including race/ethnicity (dichotomized as 1 = *white/non-Hispanic,* 0 = *other race/ethnicity*) and education status (dichotomized as 0 = *no four-year postsecondary degree*, 1 = *earned a four-year postsecondary degree*). Maternal age (in years) was obtained from patient medical records, and early pregnancy maternal body mass index (BMI, kg/m^2^) was calculated from height and weight measured at the baseline visit (< 12 weeks gestation). Mothers also reported feeding modality (i.e., breastfeeding, formula feeding, or both) at each postpartum visit; responses were categorized to indicate exclusive breastfeeding at any time (2, 6 or 12 months) postpartum.

### Analyses

Bivariate correlations between all variables of interest and potential covariates (maternal BMI, age, education, race/ethnicity, exclusive breastfeeding) were examined to determine which covariates would be included in the longitudinal models. Three longitudinal multivariate path models were estimated. Model 1 examined the relations of maternal obesogenic eating behaviors (restrained, emotional, external) and eating competence with feeding to soothe at 2 months postpartum, and the relations of feeding to soothe with infant appetitive behaviors (enjoyment of food, food responsiveness, slowness in eating, satiety responsiveness) at 6 months. Model 1 simultaneously estimated the direct effects of maternal obesogenic eating behaviors, eating competence, and feeding to soothe and the indirect effects of maternal eating behaviors and eating competence through feeding to soothe on each infant appetitive behavior. In Model 2, we added feeding to soothe at 6 months to the model. Model 2 examined all the same effects as Model 1 but added the direct effects of each infant appetitive behavior at 6 months on feeding to soothe at 6 months. Model 2 also estimated the indirect effects of maternal eating behaviors and eating competence on feeding to soothe at 6 months through feeding to soothe at 2 months and infant appetitive behaviors at 6 months. Finally, we estimated a model that replaced feeding to soothe at 6 months with feeding to soothe at 12 months (Model 3).

We used a bootstrap approach [24] to simultaneously estimate all direct and indirect effects in each of the three models. Bootstrapping maximizes power by randomly resampling the available data and repeatedly estimating a model using the random resamples. It then estimates a 95% confidence interval (CI) for the distribution of each indirect effect within the sample. The analyses specified 1000 random sampling iterations for each model. To account for any missing data across the four time points, we estimated the three models using Full Information Maximum Likelihood [25] in Mplus 8.3 [26]. Model fit was examined using the cutoff criteria (values close to or greater than 0.95 for comparative fit index [CFI] and less than 0.08 for standardized root mean square residual [SRMR]) established by Hu and Bentler [27]. Standardized estimates and confidence intervals are presented for all model results.

Sensitivity analyses were then conducted to examine whether the three models differed between infant feeding modalities. Three multiple group analyses were conducted to determine whether the relations examined in the three models differed between mothers who exclsuively breastfed their infants at any time postpartum and mothers never exculsively breasfed. Multiple group analyses are used to examine whether a model differs between two groups by comparing the chi-square fit value of a model contraining all relations to be equal between the two groups with the chi-square of the same model where all relations are allowed to vary between the two groups. A significant chi-square difference test indicates that the model does vary between the two groups; a non-significant chi-square difference test indicates that the model does not vary between the two groups.

## Results

Of the 458 women enrolled at baseline, 91 (19.9%) withdrew prior to delivery, 46 (10.0%) withdrew during postpartum, and 20 (4.4%) did not complete the measures included in this study. The final sample for the current study included 301 mother-infant dyads. Based on ANOVA, the final sample did not differ from the enrolled sample on any of the covariates (maternal age, BMI, race/ethnicity, education status), maternal eating behaviors (restrained, external, and emotional eating), maternal eating competence, feeding to soothe, or infant appetitive behaviors (enjoyment of food, food responsiveness, slowness in eating, and satiety responsiveness).

Univariate statistics and bivariate associations among all variables are presented in Table 1. Mean maternal age at baseline (< 15 weeks) was 30.5 ± 4.7 years; maternal BMI was in the normal range. Two-thirds (66.7%) of mothers were white non-Hispanic and 71.7% completed a four-year college or university degree. While maternal BMI was related to eating competence and external eating, and age was associated with restrained and emotional eating, neither maternal BMI nor age was associated with feeding to soothe or infant appetitive behaviors. Maternal education and race/ethnicity were related to infant appetitive behaviors: mothers who earned a four-year degree had infants who ate slower, and white, non-Hispanic mothers used fewer feeding to soothe behaviors at 12 months and had infants who had lower enjoyment of food. Based on these findings, only maternal education status and race/ethnicity were included as covariates in the initial models.

**Table 1:** Descriptive statistics and bivariate relations between all study variables

		1	2	3	4	5	6	7	8	9	10	11	12	13	14	15	*M*	*SD*	*n*
1.	Maternal BMI	–	–	–	–	–	–	–	–	–	–	–	–	–	–	–	27.19	6.94	458
2.	Maternal Age	−.03	–	–	–	–	–	–	–	–	–	–	–	–	–	–	30.5	4.7	458
3.	Maternal Education ^a^	−.35**	.36**	–	–	–	–	–	–	–	–	–	–	–	–	–	0.72	0.45	367
4.	Maternal Ethnicity ^b^	−.29**	.22**	.32**	–	–	–	–	–	–	–	–	–	–	–	–	0.67	0.47	381
5.	Exclusive Breastfeeding ^c^	−.25**	.07	.26**	.19**	–	–	–	–	–	–	–	–	–	–	–	0.40	0.49	311
Maternal Eating Behaviors																			
6.	Total Eating Competence	−.22**	−.03	.11	.06	.11	–	–	–	–	–	–	–	–	–	–	33.11	7.50	301
7.	Restrained Eating	−.00	.22**	.29**	.11	.07	−.08	–	–	–	–	–	–	–	–	–	2.46	0.68	312
8.	Emotional Eating	−.00	.16**	.25**	.17**	.03	−.13*	.25**	–	–	–	–	–	–	–	–	2.09	0.78	312
9.	External Eating	−.16**	.09	.18**	.06	.08	−.00	.15**	.55**	–	–	–	–	–	–	–	2.85	0.54	312
Maternal Feeding to Soothe																			
10.	2-Month	.01	.04	.01	.06	−.07	.00	.15*	.07	.18**	–	–	–	–	–	–	2.53	0.93	234
11.	6-Month	.01	−.05	.05	.07	−.03	−.01	.07	.17*	.20**	.55**	–	–	–	–	–	2.29	0.82	228
12.	12-Month	.09	−.09	−.11	−.17**	−.06	−.07	.06	.15*	.20**	.45**	.59**	–	–	–	–	2.35	0.72	276
Infant Appetitive Behaviors																			
13.	Enjoyment of Food	.10	−.12	−.11	−.13*	.09	.21**	−.15*	−.13	−.09	−.02	−.01	−.06	–	–	–	4.49	0.47	229
14.	Slowness in Eating	−.04	.03	.14*	.08	−.05	.07	.06	.06	.02	.07	.03	−.04	−.33**	–	–	2.40	0.62	229
15.	Satiety Responsiveness	.04	.02	−.02	−.05	−.12	−.09	.07	.01	.10	.08	.14*	.22**	−.26**	.16*	–	2.24	0.59	229
16.	Responsiveness to Food	.11	−.03	−.00	−.10	−.20**	−.15*	.07	.11	.14*	.17*	.25**	.25**	−.04	.16*	.04	2.23	0.65	229

^a^ 0 = no four-year degree, 1 = earned a four-year degree. ^b^ 0 = ethnic minority, 1 = white non-Hispanic. ^c^ 0 = did not exclusively breastfeed, 1 = did exclusively breastfeed

* *p* < .05, ** *p* < .01

### Model 1

#### Model fit

**Fig. 1:**
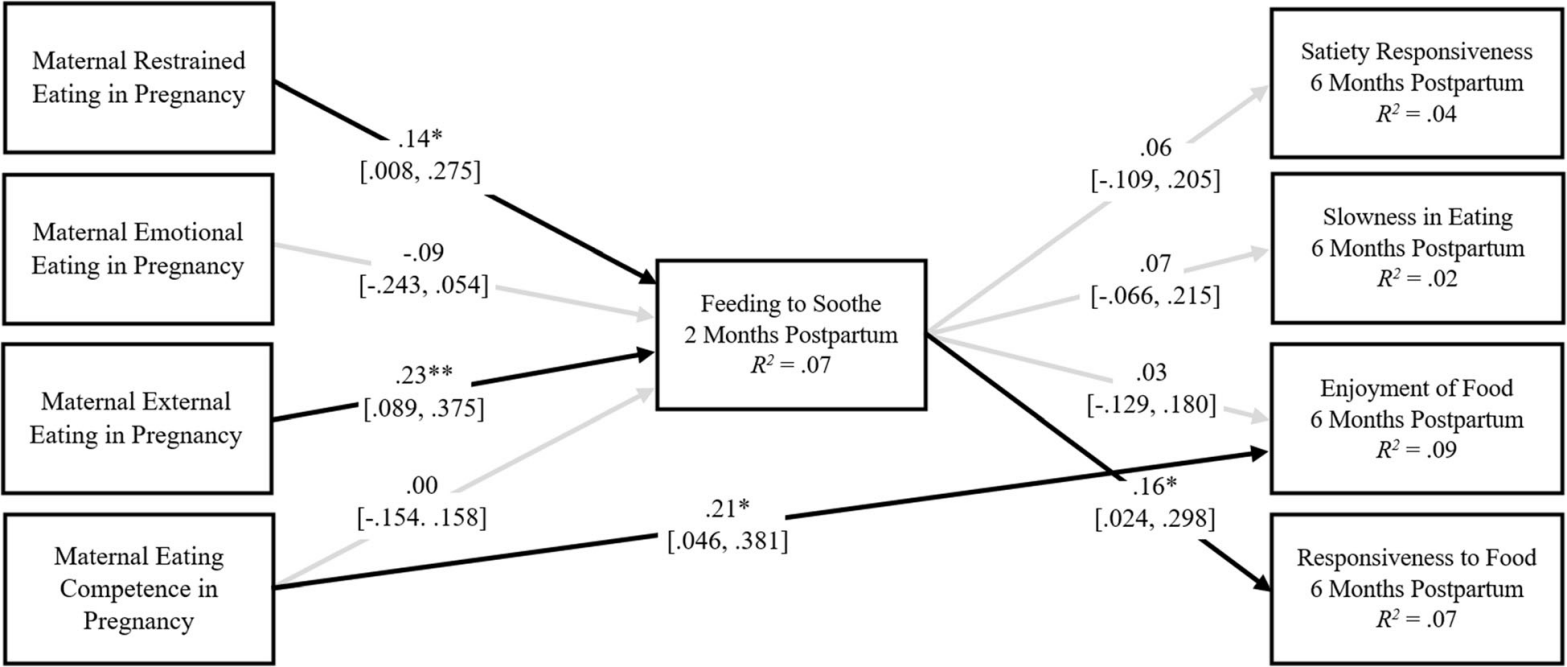
Model 1 examining the mediating effect of feeding to soothe on the relations between maternal eating behaviors, maternal eating competence, and infant appetitive behaviors. *Note*: Standardized estimates and confidence intervals are reported. Maternal ethnicity is included as a control (0 = ethnic minority, 1 = white non-Hispanic). Outcome residuals and covariances were significantly correlated, but are not shown. The direct effects of all maternal eating competence and behaviors on infant appetitive behaviors were estimated but only the significant direct effects are shown. Black arrows indicate significant relations; gray arrows indicate nonsignificant relations. *χ*^*2*^ (4) = 10.08, *p* = .04, *CFI* = .91, *TLI* = .22 *RMSEA* = .07, *SRMR* = .02. *p < .05; **p < .01

Results from Model 1, which examined the direct and indirect relations of maternal obesogenic eating behaviors with infant appetitive behaviors through feeding to soothe, are presented in Fig. 1. Initial model fit was poor (chi-square fit index [*χ*^*2*^] (5) = 16.08, *p* = .01, *CFI* = .83, Tucker-Lewis index [*TLI*] = −.37, root mean square error of approximation [*RMSEA*] = .09, *SRMR* = .02), but improved substantially when only maternal race/ethnicity was included as a covariate (*χ*^*2*^ (4) = 10.08, *p* = .04, *CFI* = .91, *TLI* = .22, *RMSEA* = .07, *SRMR* = .02). Therefore, only maternal race/ethnicity was included as a covariate in all three longitudinal models.

#### Direct effects

Greater maternal restrained eating and external eating were related to more frequent feeding to soothe at 2 months, and greater maternal eating competence was related to greater infant enjoyment of food at 6 months. More frequent feeding to soothe at 2 months was related to greater infant food responsiveness at 6 months.

#### Indirect effects

Indirect effects were observed of greater maternal restrained eating (*β* = .022; 95% CI [.002, .068]) and external eating (*β* = .037; 95% CI [.005, .092]) on greater infant food responsiveness through more frequent feeding to soothe.

### Model 2

#### Model fit

**Fig. 2:**
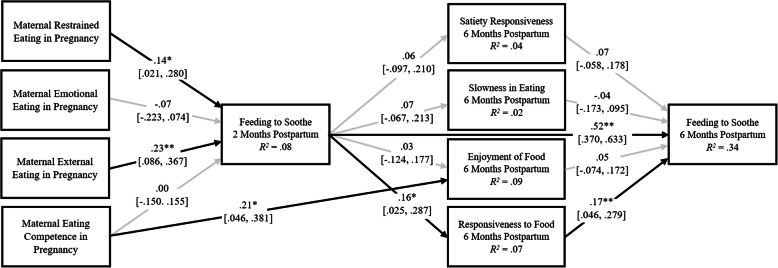
Model 2 examining the reciprocal associations of feeding to soothe at 2 and 6 months and infant appetitive behaviors. *Note*: Standardized estimates and confidence intervals are reported. Maternal ethnicity is included as a control (0 = ethnic minority, 1 = white non-Hispanic). Outcome residuals and covariances were significantly correlated, but are not shown. The direct effects of all maternal eating competence and behaviors on infant appetitive behaviors were estimated but only the significant direct effects are shown. Black arrows indicate significant relations; gray arrows indicate nonsignificant relations. *χ*^*2*^ (9) = 22.43, *p* = .01, *CFI* = .91, *TLI* = .54, *RMSEA* = .07, *SRMR* = .03. *p < .05; **p < .01

The results from Model 2, which examined the reciprocal relations between infant appetitive behaviors at 6 months and feeding to soothe at 2 and 6 months, indicated adequate fit (*χ*^*2*^ (9) = 22.43, *p* = .01, *CFI* = .91, *TLI* = .54, *RMSEA* = .07, *SRMR* = .03; Fig. 2).

#### Direct effects

All direct effects observed in Model 1 were also significant in Model 2. Additionally, more frequent feeding to soothe at 2 months was related to more frequent feeding to soothe at 6 months, and greater infant responsiveness to food at 6 months was related to more frequent feeding to soothe at 6 months.

#### Indirect effects

Greater maternal restrained eating (*β* = .023; 95% CI [.003, .070]) and external eating (*β* = .036; 95% CI [.006, .091]) were indirectly related to greater infant responsiveness to food at 6 months through feeding to soothe at 2 months, while greater maternal restrained eating (*β* = .004; 95% CI [.001, .013]) and external eating (*β* = .006; 95% CI [.001, .020]) were also related to more frequent feeding to soothe at 6 months through more frequent feeding to soothe at 2 months and then through greater infant food responsiveness at 6 months. Additionally, more frequent feeding to soothe at 2 months was related to more frequent feeding to soothe at 6 months through greater infant responsiveness to food at 6 months (*β* = .026; 95% CI [.006, .065]).

### Model 3

#### Model fit

**Fig. 3:**
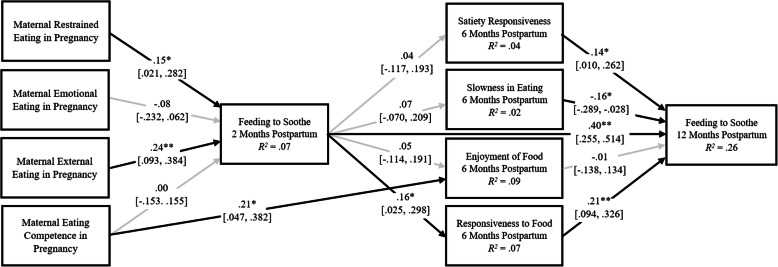
Model 3 examining the reciprocal associations of feeding to soothe at 2 and 12 months and infant appetitive behaviors. *Note*: Standardized estimates and confidence intervals are reported. Maternal ethnicity is included as a control (0 = ethnic minority, 1 = white non-Hispanic). Outcome residuals and covariances were significantly correlated, but are not shown. The direct effects of all maternal eating competence and behaviors on infant appetitive behaviors were estimated but only the significant direct effects are shown. Black arrows indicate significant relations; gray arrows indicate nonsignificant relations. *χ*^*2*^ (9) = 21.83, *p* = .01, *CFI* = .90, *TLI* = .51, *RMSEA* = .07, *SRMR* = .03. *p < .05; **p < .01

Results from Model 3, which examined the reciprocal relations between infant appetitive behaviors at 6 months and feeding to soothe at 2 and 12 months, indicated adequate fit (*χ*^*2*^ (9) = 21.83, *p* = .01, *CFI* = .90, *TLI* = .51, *RMSEA* = .07, *SRMR* = .03; Fig. 3).

#### Direct effects

All direct effects found in Models 1 and 2 were also significant in Model 3. Additionally, greater infant satiety responsiveness and faster infant eating speed at 6 months were related to more frequent feeding to soothe at 12 months.

#### Indirect effects

Similar indirect effects present in Model 2 were significant in Model 3. Greater maternal restrained eating (*β* = .023; 95% CI [.003, .068]) and external eating (*β* = .038; 95% CI [.006, .091]) were related to greater infant responsiveness to food at 6 months through more feeding to soothe at 2 months. Greater maternal restrained eating (*β* = .005; 95% CI [.001, .016]) and external eating (*β* = .008; 95% CI [.002, .023]) were also related to more frequent feeding to soothe at 12 months through more frequent feeding to soothe at 2 months and then through greater infant food responsiveness at 6 months. Additionally, more frequent feeding to soothe at 2 months was related to more frequent feeding to soothe at 12 months through greater infant responsiveness to food at 6 months (*β* = .033; 95% CI [.009, .076]).

### Sensitivity analyses

Results from the multiple group analyses indicated that there were no differences between mothers who exclusively breastfed their infants at any point postpartum versus mothers who did not for the relations among maternal eating behaviors, feeding to soothe, and infant appetitive behaviors (Model 1 [*χ*^*2*^_*diff*_ (29) = 14.35, *p* = .99], Model 2 [*χ*^*2*^_*diff*_ (34) = 21.88, *p* = .95], and Model 3 [*χ*^*2*^_*diff*_ (34) = 20.23, *p* = .97]).

## Discussion

In this study examining the relations between maternal eating behaviors, feeding to soothe, and infant appetitive behaviors, greater maternal restrained and external eating were related to greater infant responsiveness to food indirectly through more feeding to soothe; however, maternal eating competence was directly related to more infant enjoyment of food and unrelated to feeding to soothe. These relations persisted in the models investigating the reciprocal relations between feeding to soothe and infant appetitive behaviors. More frequent feeding to soothe at 2 months predicted greater responsiveness to food at 6 months, and greater responsiveness to food at 6 months was related to more frequent feeding to soothe at 6 months. Further, faster eating speed, greater satiety responsiveness, and greater responsiveness to food were related to more frequent feeding to soothe at 12 months. Together, these results highlight the complex relations between maternal eating behaviors, feeding to soothe, and infant appetitive behaviors.

These relations in infancy are consistent with prior studies that found more frequent feeding to soothe was related to more obesogenic eating behaviors in toddlers and older children [9, 10]. Findings underscore the impact of emotionally driven feeding on appetitive behaviors in childhood and indicate that maternal feeding practices may influence the development of obesogenic eating as early as the first year of life. Our study also expands on previous work by providing preliminary support for potential bidirectional relations between feeding to soothe and infant appetitive behaviors. More feeding to soothe early in infancy (i.e., 2 months) is associated with more obesogenic child appetitive behaviors, and those appetitive behaviors then relate to more feeding to soothe later in infancy (6 months and 12 months). Feeding in response to infant emotional cues may acclimate children to eating in response to those cues rather than eating only in response to hunger. As infants acclimate to eating in response to non-hunger cues, they may then develop obesogenic eating behaviors, such as a high responsiveness to food, which then encourages more feeding to soothe behaviors from their mothers [13].

Findings from this study illustrate the complex relations between maternal eating behaviors, feeding to soothe, and infant appetitive behaviors. Partially consistent with prior work indicating that maternal emotional and restrained eating were related to emotional and instrumental feeding styles in 3–5 year-old children, respectively [18], we found that more maternal restrained and external eating were related to more frequent feeding to soothe, but maternal emotional eating was not related to feeding to soothe. These differences could be due to differing ages of the sample or the study design (cross-sectional versus longitudinal). Additionally, the modest effects sizes observed suggest the presence of additional environmental and biological influences on feeding to soothe and appetitive behaviors.

Our findings that both external and restrained eating were indirectly related to infant responsiveness to food through more frequent feeding to soothe extend prior research [15, 16] by examining the mechanism through which maternal eating behaviors are related to child eating behaviors. Findings suggest that mothers may transmit their expectations and emotions around eating to their child through their feeding behaviors; that is, mothers with greater obesogenic eating behaviors acclimate their child to similarly unhealthy eating behaviors [18] by feeding the child in response to distress or emotions rather than in response to hunger [28]. Additionally, although maternal eating competence was not related to feeding to soothe, greater eating competence was directly related to greater infant enjoyment of food. Mothers with greater eating competence may perceive that their infants enjoy food more than those with lower eating competence.

This study underscores the need to account for the complex relations between maternal eating behaviors and feeding to soothe in the prevention of child obesogenic appetitive behaviors. Our findings support intervention approaches that instruct parents in appropriate feeding behaviors (e.g., feeding in response to hunger rather than in response to negative emotions) to promote healthy eating in infants (e.g., [29]). Intervention effectiveness may additionally be enhanced by addressing parent obesogenic and competent eating behaviors and promoting recognition of how these may influence feeding behaviors. Future studies may also wish to examine whether there are bidirectional relations of feeding to soothe and appetitive behaviors with infant weight.

Study findings should be interpreted in light of its strengths and limitations. Although this study was observational, the longitudinal design strengthens internal validity and enables an examination of temporal precedence. The use of bootstrapping to estimate the indirect effects provides higher power than other estimates of indirect effects and does not require assumptions of normality in the data [30], thus allowing detection of modest effects. Although the Baby Eating Behaviors Questionnaire has been validated across samples from numerous populations, the low internal consistency of the satiety responsiveness subscale could indicate that this measure does not reflect a distinct construct in our sample. Additionally, the single measurement of appetitive behaviors at 6 months precludes the ability to fully examine bidirectional relations between feeding to soothe and appetitive behaviors, and all measures came from maternal reports, which could artificially inflate the relations. Also, although the sample consisted of nearly 30% minority race/ethnic mothers, the single geographic region and relatively high education level of the sample limit generalizability. Finally, this study did not account for the feeding behaviors of other caregivers in the household, which may modify the relationship of maternal feeding to soothe with infant appetitive behaviors.

## Conclusions

Findings from this longitudinal prospective cohort study of 301 mother-infant dyads in North Carolina indicate that maternal eating behaviors are directly and indirectly related to infant appetitive behaviors. Additionally, these results provide preliminary evidence that the relations between feeding to soothe and infant appetitive behaviors may be bidirectional. These results underscore the need to examine how parental feeding behaviors are influenced both by parental eating behaviors and child appetitive behaviors throughout infancy and childhood. Additionally, these findings suggest that maternal influences on appetitive behaviors are evident as early as the first year of life.

## Data Availability

The datasets used and/or analyzed during the current study are available from the corresponding author on reasonable request. Following publication of study objectives, de-identified data will be shared in the NICHD Data and Specimen Hub.

## Abbreviations

PEAS: Pregnancy Eating Attributes Study
CFI: Comparative fit index
TLI: Tucker-Lewis index
RMSEA: Root mean square error of approximation
SRMR: Standardized root mean square residual
